# Robust Image Restoration for Motion Blur of Image Sensors

**DOI:** 10.3390/s16060845

**Published:** 2016-06-09

**Authors:** Fasheng Yang, Yongmei Huang, Yihan Luo, Lixing Li, Hongwei Li

**Affiliations:** 1 Institute of Optics and Electronics, Chinese Academy of Sciences, P.O. Box 350, Shuangliu, Chengdu 610209, China; yangfs_ioe@163.com (F.Y.); luo.yihan@foxmail.com (Y.L.); icecliff@126.com (L.L.); zkygds@126.com (H.L.); 2 Key Laboratory of Optical Engineering, Chinese Academy of Sciences, Chengdu 610209, China; 3 University of Chinese Academy of Sciences, 19 A Yuquan Rd, Shijingshan District, Beijing 100039, China

**Keywords:** blind image restoration, blur kernel estimation, edge selection, image sensor signal processing, motion deblurring

## Abstract

Blind image restoration algorithms for motion blur have been deeply researched in the past years. Although great progress has been made, blurred images containing large blur and rich, small details still cannot be restored perfectly. To deal with these problems, we present a robust image restoration algorithm for motion blur of general image sensors in this paper. Firstly, we propose a self-adaptive structure extraction method based on the total variation (TV) to separate the reliable structures from textures and small details of a blurred image which may damage the kernel estimation and interim latent image restoration. Secondly, we combine the reliable structures with priors of the blur kernel, such as sparsity and continuity, by a two-step method with which noise can be removed during iterations of the estimation to improve the precision of the estimated blur kernel. Finally, we use a MR-based Wiener filter as the non-blind deconvolution algorithm to restore the final latent image. Experimental results demonstrate that our algorithm can restore large blur images with rich, small details effectively.

## 1. Introduction

**Figure 1:**
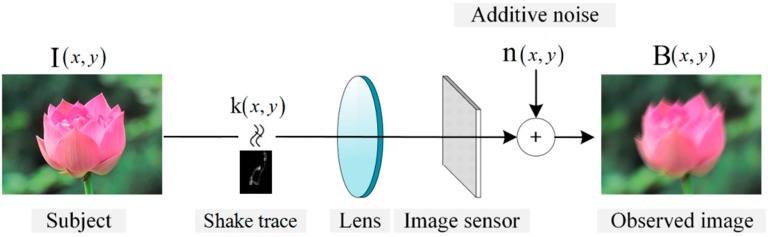
Image degradation model for motion blur of image sensor.

Motion blur widely exists in digital photography and leads to disappointing blurry images with inevitable information loss. Due to the mechanism of image sensors that integrate incoming lights for an amount of time to produce images, if a relative motion happens between the subject and the image sensors during the integration time, a blurred image will be produced as shown in Figure 1.

Motion deblurring has been deeply researched in computer vision and image processing due to its involvement of many challenges in problem formulation, regularization, and optimization. The motion blur produced by camera shake is generally modeled as:

(1) B = I⊗k + n

where *B*, *I*, *k*, and *n* represent the blurred image, latent sharp image, blur kernel (also known as point spread function, PSF), and additive noise, respectively. ⊗ is the convolution operator. The blur kernel delineates motion trace between the subject and image sensors. Generally, the size of the blur kernel is relatively smaller than that of the latent sharp image and its value is non-negative. Then, estimating the latent sharp image *I* and blur kernel *k* from the blurred image *B* is a severely ill-conditioned inverse problem, which requires regularization to alleviate its ill-conditionness and stabilize the solution.

Single-image motion debluring can be roughly categorized into two groups: non-blind deconvolution and blind deconvolution. In non-blind deconvolution, both the blurred image and blur kernel are known and the latent sharp image is restored from the blurred image using the blur kernel. Early famous algorithms such as Richardson-Lucy (R.L.) [1] and Weiner filter [2] which are simple and efficient are still widely used. However, these algorithms may produce ringing artifacts near strong edges inevitably due to the drawback of being sensitive to noise. Recently, some robust motion deblurring algorithms have been proposed [3,4,5,6].

In blind deconvolution, the blur kernel is unknown and restoring the latent sharp image becomes more challenging. Most blind deconvolution algorithms commonly perform with some estimation criterions, such as maximum *a posteriori* (MAP) or Bayesian, and some priors of the latent sharp image or blur kernel to restore them from the blurred image. Previous algorithms, firstly, impose constraints on the motion blur kernel, such as sparse prior [3], Gaussian prior [7], or color prior [8]. After obtaining the blur kernel, these algorithms [3,7,8] restore the latent sharp image by non-blind deconvolution approaches.

In this paper, we introduce a robust blind motion restoration algorithm estimating the blur kernel and latent sharp image iteratively and hierarchically. As is reported [7], only reliable structures can contribute to kernel estimation, we firstly construct a pyramid representation of the blurred image by down-sampling with factor of $\sqrt{2}$ and extract the reliable structures according to the characteristics of the blurred image in each level. Then we estimate the blur kernel and latent sharp image in each level alternately. In order to eliminate the damage of noise to kernel estimation, which further conduce unreliable deblurred results, we combine some priors of the blur kernel with the reliable structures of the blurred image to estimate the blur kernel. For latent sharp image estimation, we add a spatial prior to suppress the noise and ringing artifacts. After obtaining the blur kernel and latent sharp image in a coarse level, we up-sample them to a finer level and reiterate the process above until reaching the finest level.

The contributions of this paper can be listed as follows:

1. In order to eliminate the effect of noise and ambiguous structures which may damage kernel estimation, we propose a novel structure extraction method with which the reliable structures can be selected adaptively and effectively;
2. As motion blur kernel is sparse and delineates the motion trace between the subject and image sensors, we introduce a two-step method for the kernel estimation process to eliminate the noise and guarantee the sparsity and continuity.

The rest of this paper is organized as follow: in Section 2 we review related works, the proposed kernel estimation and non-blind deconvolution algorithm are described in Section 3, experimental results are illustrated in Section 4 and, finally, we conclude our algorithm in Section 5.

## 2. Related Work

Blurred image restoration is a fundamental problem in enhancing images acquired by various types of image sensors [9,10,11,12]. Although various image sensors’ signal processing techniques have been proposed, restoration of blurred images modeled in Equation (1) is still a challenging task because of the latent sharp image and blur kernel are highly unconstrained and there is no unique combination of them whose convolution is equal to the blurred image. Previous works generally impose constraints on the blur kernel and represent it in a simple parametric form. However, as is shown [3], the real blur kernels are too complicated to be formulated in a simple parametric form. Fergus *et al.* [3] used a zero-mean Mixture of Gaussian to approximate the gradients of nature image and proposed a variational Bayesian inference algorithm to deblur images. Shan *et al.* [4] used a series of optimization techniques to avoid trivial solutions and so be robust to noise. Krishnan *et al.* [13] introduced a new normalized sparsity as the regularization term into their MAP framework to estimate the blur kernel. Xu *et al.* [14] introduced an image decomposition scheme to make image deblurring process more robust. Levin *et al.* [15] showed the limitation of the naive MAP approach and suggested estimating the MAP of the blur kernel alone while marginalizing over the latent sharp image in [16]. Oh *et al.* [17] adopted a piecewise-linear model to approximate the curves for the blur kernel estimation. Shao *et al.* [18] impose a type of non-stationary Gaussian prior on the gradient fields of sharp images to estimate the blur kernel. Although extensive works have been done, the estimated kernels still contain some noise, and selecting a kernel with a hard threshold will destroy the intrinsic structures of the blur kernel.

Another group of algorithms [6,7,8,19] use predicted sharp edges to estimate the blur kernel. Money and Kang [19] used a shock filter to sharpen edges. Joshi *et al.* [8] used edge profiles instead of shock filtering, but those methods only work for small blurs. Cho and Lee [6] combined a bilateral filter with the shock filter to predict sharp edges from the blurred image iteratively. However, as described in the paper, that method could not predict sharp edges correctly for large blurs. Xu and Jia [7] introduced a mask to select useful gradients of the blurred image for kernel estimation. Although the performance improved greatly, the continuity and sparsity of the blur kernels still cannot be guaranteed and the estimated kernels still occasionally contain some noise.

With a known blur kernel, the blind deconvolution problems can be solved by the non-blind deconvolution approaches. However, as the latent sharp image restoration is very sensitive to noise and may produce some undesirable artifacts, the non-blind deconvolution is still ill-conditioned. Many works [4,20,21] have been conducted to overcome this problem. Levin *et al.* [20] used a heavy-tailed function to alleviate the ringing artifacts, which was based on the model of sparse image derivatives distribution. Yuan *et al.* [21] proposed a multi-scale bilateral Richardson-Lucy algorithm to reduce ringing artifacts. Shan *et al.* [4] adopted a local smoothness prior to suppress the artifacts in smooth regions.

## 3. Single Image Blind Deconvolution Using Reliable Structure

**Figure 2:**
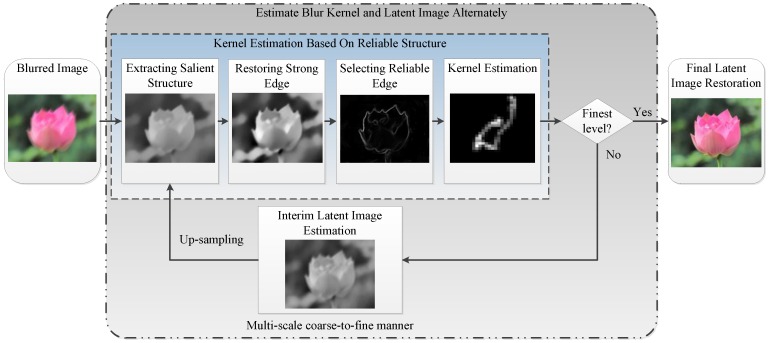
The flowchart of the proposed blind deconvolution algorithm.

**Figure 3:**
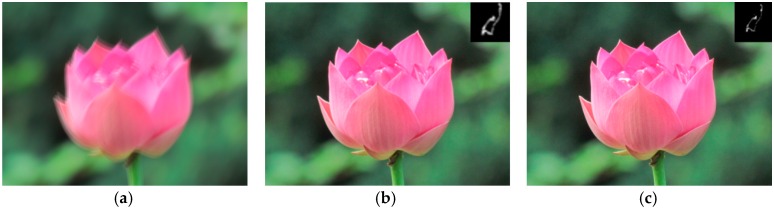
The restoration of a synthetic blurred image. (**a**) blurred image; (**b**) restored image and estimated blur kernel; and (**c**) ground truth image and blur kernel.

Many algorithms have been proposed to restore the latent sharp image using image structures and the deblurred results depend much on the reliability of the extractive structures. We propose a new method to extract the reliable structures, which will be discussed in Section 3.1. The blur kernel estimated by existing methods usually contain some noise which may damage the latent sharp image estimation. We introduce a two-step method which can guarantee the continuity and sparsity of the blur kernel to eliminate the noise in Section 3.2. The latent sharp image restoration method will be discussed in Section 3.3 and Section 3.4. And we give the multi-scale implementation of our algorithm in Section 3.5. The overview framework of our approach is shown in Figure 2, and the deblurred result of a synthetic blurred image is shown in Figure 3.

### 3.1. Reliable Structure Extraction from Blurred Image

As is discussed in [7], the structures of the blurred image do not always improve the blur kernel estimation. On the contrary, the edges whose size are smaller than that of the blur kernel will deteriorate the blur kernel estimation. Inspired by the total variation-based noise removal algorithm [22], we treat the textures and small details as “noise” and introduce a salient structure extraction method to separate the helpful structures of the blurred image for the blur kernel estimation from the detrimental textures and small details. The energy function is formulated as follow:

(2) $$E\left(I_{s}\right)\;=\;\frac{1}{2\theta M(B)}{\left\|I_{s}\;-\;I\right\|}_{2}^{2}\;+\;{\left\|I_{s}\right\|}_{TV2}$$

where ${\left\|I_{s}\right\|}_{TV2}$ is the isotropic TV norm of salient structure *I*_s_ defined as Equation (3), which can preserve edges.

(3) $${\left\|I_{s}\right\|}_{TV2}=\int_{p\in I_{s}}\sqrt{\partial x{I_{sp}}^{2}\;+\;\partial y{I_{sp}}^{2}}dp$$

where *M* is the gradient attenuation function [23] defined as Equation (4), which is a self-adaptive weight for textures and small details attenuation:

(4) $$M\left(L\right)\;=\;\frac{\alpha_{s}}{\left\|\nabla L\left(x,y\right)\right\|}{\left(\frac{\left\|\nabla L\left(x,y\right)\right\|}{\alpha_{s}}\right)}^{\beta }$$

where *L* is the image to be measured and the parameter, $\alpha_{s}$ controls the gradient magnitudes which remain unchanged, and β determines how much the lager magnitude will be attenuated (assuming β < 1), while gradients of magnitude smaller than $\alpha_{s}$ are slightly magnified. As *M*(B) is the self-adaptive weight in Equation (2), the salient structures will be kept more while the flat areas and narrow strips will be smoothed, so the salient structure *I*_s_ can be obtained by minimizing Equation (2). We set the coarsest version of the blurred image *B* as the initial value of the latent image *I* for Equation (2).

**Figure 4:**
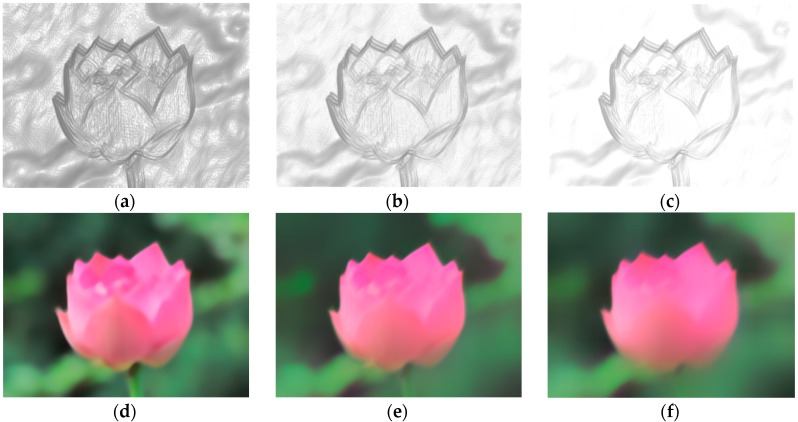
The influence of $\alpha_{s}$ on extracted salient structure. (**a**) *s* = 0.2; (**b**) *s* = 0.5; (**c**) *s* = 0.8; (**d**) extracted salient structure relevant to *s* = 0.2; (**e**) extracted salient structure relevant to *s* = 0.5; and (**f**) extracted salient structure relevant to *s* = 0.8.

We empirically set β between 0.8 and 0.9, and $\alpha_{s}$ to *s* times the average gradient magnitude of the blurred image in each level. Figure 4 show the self-adaptive weight *M*(B) with different parameters and the corresponding extracted salient structures, a larger $\alpha_{s}$ will give a strong attenuation to the smooth regions while a smaller $\alpha_{s}$ will preserve more details.

After obtaining the salient structure *I*_s_, we enhance it by the the shock filter [24] to recover strong edges:

(5) $$\partial {\tilde{I}}_{s}/\partial t\;=\;-sign\left(\Delta I_{s}\right){\left\|\nabla I_{s}\right\|}_{2}$$

where t is the evolution time of the shock filter, $\Delta I_{s}$ and $\nabla I_{s}$ are the Laplacian and gradient of *I*_s_, respectively. The enhanced structure ${\tilde{I}}_{s}$ contains not only the sharp edges but also the enhanced noise. In order to remove the noise, we use a mask to select reliable structure S, which will be used to estimate the blur kernel:

(6) $$\nabla S\;=\;\nabla {\tilde{I}}_{s}H\left(\tau \;-\;M(I_{s})\right)$$

where *H*(·) denotes the Heaviside step function which outputs ones for positive values and zeros otherwise, and *M* is defined as Equation (4). As *M*(*I*_s_) is large in smooth regions, while small near strong edges, and *M*(${\tilde{I}}_{s}$) is small near not only strong edges, but also enhanced noise, one can set an appropriate value of the threshold τ, referring to the value of *M*(${\tilde{I}}_{s}$) near strong edges and the differences between the value of *M*($I_{s}$) and *M*(${\tilde{I}}_{s}$) in smooth regions to eliminate the noise and obtain the reliable structures.

It is known that the less the salient edges are used in kernel estimation, the more unreliable the estimated blur kernel is. We take the following strategies to guarantee the reliability of the estimated blur kernel:

Firstly, as the initial value of τ is critical to kernel estimation [7], we take the method of [6] to set the value of τ adaptively at the beginning of the iterative restoration process. Four directions of the image gradients are taken into account to guarantee enough information of the salient edges are used to estimate the blur kernel. Additionally, the value of τ for later iterations is set to allow that at least $0.5\sqrt{P_{I}P_{k}}$ pixels take part in the kernel estimation in each group. $P_{k}$ and $P_{I}$ are the total number of pixels of the blur kernel and input image, respectively.

Secondly, as more edges are needed to estimate the blur kernel in higher level of the pyramid, the parameters θ and $\alpha_{s}$ are decreased to bring more edge information into the kernel estimation. Our strategies allow the recovery of fine structures during kernel refinement.

**Figure 5:**
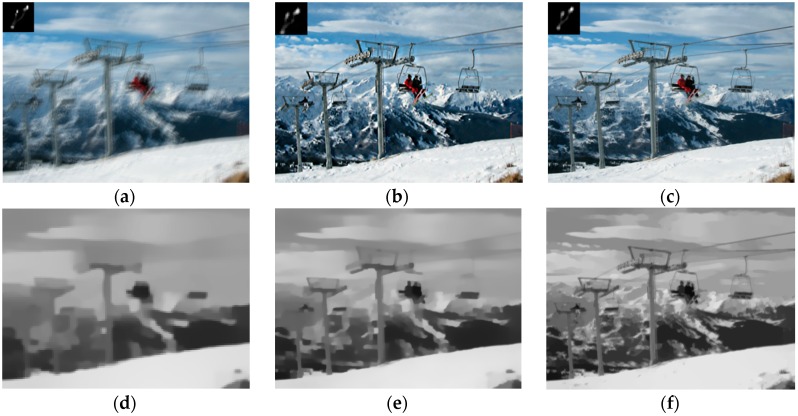
Results comparison of [7] and our method. (**a**) Blurred image and truth kernel; (**b**) result of [7]; (**c**) result of our method; and (**d**–**f**) interim ∇S maps with our method. Our result shown in (**c**) is better.

Figure 5d–f show some interim ∇S maps in different levels. It is obvious that the higher the level is, the more the sharp edges participate in kernel estimation.

### 3.2. Kernel Estimation and Refinement

As motion blur ascribes to the relative motion between the subject and image sensors within the exposure time period, the blur kernel delineates the motion trace between them and should be continuous and sparse. We employ a two-step method to guarantee the sparsity and continuity, respectively.

**Estimation**. We combine the strictly-selected edges ∇S with a Hyper-Laplacian prior regularization term by the MAP estimation criterion to estimate the blur kernel with sparsity. The energy function is formulated as follow:

(7) $$E\left(k\right)\;=\;\sum_{S_{*,}B_{*}}\omega_{*}{\left\|S_{*}⊗k\;-\;B_{*}\right\|}_{2}^{2}\;+\;\zeta {\left\|k\right\|}_{\gamma }^{\gamma }$$

(8) $$\mathrm{s.t.}\;k\left(x,\;y\right)\;\geq \;0,\;\sum_{\left(x,y\right)}k\left(x,\;y\right)\;=\;1$$

where $\omega_{*}$ is the weight for each partial derivative, ζ is the weight for the Hyper-Laplacian regularization term with 0 < γ < 1. $S_{*}$ and $B_{*}$ are the partial derivatives of the selected reliable structures and blurred image.

(9) $$\left(S_{*},\;B_{*}\right)\in \{\left(\nabla S_{x},\;\partial_{x}B\right),\;\left(\nabla S_{y},\;\partial_{y}B\right),\;\left(\partial_{x}\nabla S_{x},\;\partial_{xx}B\right),\;\left(\partial_{y}\nabla S_{y},\;\partial_{yy}B\right)\}$$

We run the constrained iterative reweighted least square (IRLS) method [14] two iterations to minimize Equation (7) and use CG method for the inner IRLS system.

**Figure 6:**
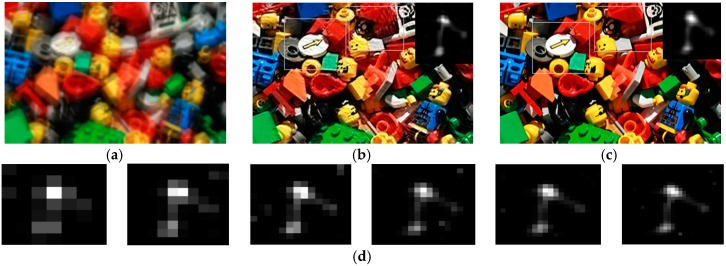

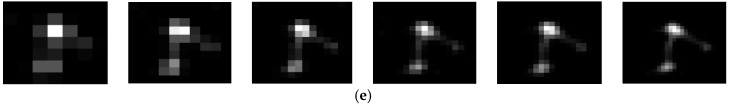
Comparison of results with and without the refinement step using same selected ∇S maps. (**a**) Blurred image; (**b**) estimated sharp image and blur kernel without the refinement step; (**c**) estimated sharp image and blur kernel with the refinement step; (**d**) show the iterations of the blur kernel estimation without the refinement step; and (**e**) show the iterations of the blur kernel estimation with the refinement step. Without the refinement step, the estimated blur kernel contains some noise which may deteriorate the deblurred image. Restored image in (c) and blur kernel in (e) outperform the results in (b,d).

**Refinement**. As the estimated blur kernel may contain some noise which may deteriorate the following the estimated interim latent images and kernels (e.g., the deblurred image and estimated kernel shown in Figure 6), we eliminate the noise by checking the pixels’ continuity of the estimated blur kernel. The energy function is defined as follow, which can be minimized using the alternative optimization method [25].

(10) $$E\left(\tilde{k}\right)\;=\;\sum_{p\in \tilde{k}}{\left({\tilde{k}}_{p}\;-\;k_{p}\right)}^{2}\;+\;\lambda C\left(\tilde{k}\right)$$

where λ is the weight for the continuity constrained term which is defined as follow:

(11) $$C\left(\tilde{k}\right)\;=\;\{p|\left|\partial_{x}{\tilde{k}}_{p}\right|\;+\;\left|\partial_{y}{\tilde{k}}_{p}\right|\;\neq \;0\}$$

During the kernel estimation process, the two steps above are taken alternately three times (Itr = 3) with γ = 0.5, empirically, in each level of the pyramid. The value of λ can be set refer to the size of the blur kernels. Algorithm 1 illustrates the blur kernel estimation algorithm.
**Algorithm 1.** Blur Kernel Estimation. **Input**: Blurred image, reliable structures and the initial value of *k* from previous iterations or previous level;  **for**
*n* = 1 to Itr (Itr: number of iterations) **do**  Estimate the blur kernel using the reliable structures. (Equation (7))  Refine the blur kernel by solving Equations (10) and (11) alternately.  $k\leftarrow \tilde{k}$; **end for**  **Output:** Blur kernel *k*.

**Figure 7:**
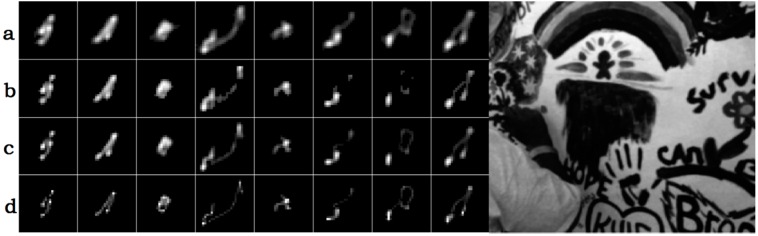
Comparison of the estimated kernels. (**a**) results of Fergus *et al.* [3]; (**b**) results of Xu and Jia [7]; (**c**) our results; and (**d**) the ground truth blur kernels.

**Figure 8:**
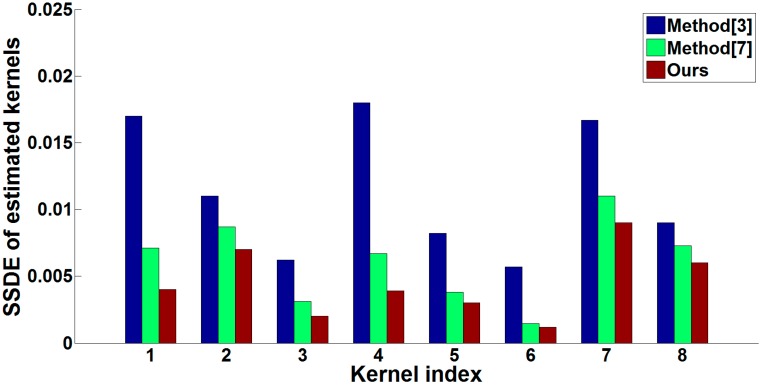
Comparison of the estimated kernels in terms of SSDE.

We use the dataset of [15] to testify the effectiveness of our kernel estimation method, and the comparison of the estimated kernels between some previous works and our algorithm is illustrated in Figure 7. Benefiting from both the reliable structures and two-step estimation method, our kernel estimation results perform better. Then we adopt the SSDE (sum of squared differences error, which is defined as Equation (12)) to evaluate the accuracy of the estimated kernels shown in Figure 7 and give the results comparison in Figure 8.

(12) $$SSDE\;=\;{\sum_{\left(x,y\right)}\left[k_{GT}(x,y)\;-\;k(x,y)\right]}^{2}$$

where k and $k_{GT}$ denote the estimated blur kernel and ground truth blur kernel respectively.

### 3.3. Interim Latent Image Restoration

As attention is paid on recovering the salient edges during the interim latent image restoration process, we use the strictly selected edges ∇S as a spatial prior to restore the coarse version of the latent image. We minimize the following energy function to obtain the latent image:

(13) $$E\left(L\right)\;=\;{\left\|L⊗k\;-\;B\right\|}_{2}^{2}\;+\;\kappa {\left\|\nabla L\;-\;\nabla S\right\|}_{2}^{2}$$

where κ is the weight for the spatial prior regularization term which can suppress the noise and avoid ringing artifacts. We employ FFTs on Equation (13) and take the partial derivative with respect to *k* to zero. The closed-form of the latent image is given by Equation (14):

(14) $$L\;=\;F^{-1}\left(\frac{\bar{F\left(k\right)}\cdot F\left(B\right)\;+\;\kappa \left(\bar{F\left(\partial_{x}\right)}\cdot F\left(\nabla S_{x}\right)\;+\;\bar{F\left(\partial_{y}\right)}\cdot F\left(\nabla S_{y}\right)\right)}{F{\left(k\right)}^{2}\;+\;\kappa \left(F{\left(\partial_{x}\right)}^{2}+F{\left(\partial_{y}\right)}^{2}\right)}\right)$$

where *F* and $F^{-1}$ denote the forward and inverse Fourier transforms, respectively, and $\bar{F\left(*\right)}$ is the complex conjugate of F(∗).

### 3.4. Final Non-Blind Deconvolution

After obtaining the blur kernel, the final latent image will be restored from the full-scale blurred image, which contains more noise and the process is time consuming. In order to achieve high robustness and processing speed, we adopt the MR-based Wiener filter [26] to restore the final latent sharp image in frequency domain, whose transfer function is formulated as follow:

(15) $$F\left(L\right)\;=\;\frac{\bar{F(k)}\cdot F\left(B\right)}{{\left|F(k)\right|}^{2}\;+\;\Gamma }$$

where Γ is defined as:

(16) $$\Gamma \;=\;{\left|F\left(k\right)\left(u_{MH},0\right)\right|}^{2\eta }\cdot {\left[\underset{u\in D_{T}}{\max}\left|F\left(k\right)\left(u,0\right)\right|\right]}^{2\left(1-\eta \right)}\;-\;{\left[\underset{u\in D_{T}}{\min}\left|F\left(k\right)\left(u,\;0\right)\right|\right]}^{2}$$

The symbols $D_{T}$ and $u_{MH}$ denote the cut-off frequency and boundary between the high-frequency and mid-frequency regions of the blur kernel frequency spectrum F(k)(u, v), respectively, and (u, v) is the index in frequency domain. The values of them are calculated with the method proposed in that paper. The parameter η controls the compromise between image details recovery and noise suppression, and we set η = 0.8 in our experiment.

### 3.5. Multi-Scale Implementation

In order to deal with large blur kernels and make the restoration algorithm effectively and efficiently, we build a coarse-to-fine pyramid of images with a down-sampling factor of $\sqrt{2}$ to estimate the blur kernel in multi-scale resolution. The number of pyramid levels is determined by the size of the blur kernel such that the width or height of the blur kernel at the coarsest level is about 3–7 pixels. The blur kernel and interim latent image are estimated alternately for a few iterations at each level. After obtaining the full scale blur kernel, the final latent sharp image is restored by using a MR-based Wiener filter. Algorithm 2 outlines our approach.
**Algorithm 2.** Overall Algorithm. **Input:** Blurred image *B*, parameters θ, $\alpha_{s}$ and the size of blur kernel;  Build an image pyramid {*B_s_*} and all-zero kernel pyramid {*k_s_*} with level index {1, 2, …, *n*} according to the size of blur kernel;  1. Blind estimation of blur kernel  **for** = 1 to *n*
**do**   Compute adaptive weight *M*(*B_s_*) (Equation (4)).   **for**
*i* = 1 to *m* (m iterations) **do**    Extract salient structure *I_s_* (Equation (2)).    Select reliable structure ∇S for kernel estimation (Equation (6))    Estimate blur kernel according to Algorithm 1.    Restore interim latent image *L* (Equation (14))     θ←θ/1.1, $\alpha_{s}\leftarrow \alpha_{s}/1.1$   **end for**   Up-sample latent image: $L_{l+1}\leftarrow L_{l}↑$.   Porject $k_{l}$ onto the constraints (Equation (8)) and up-sample blur kernel: $k_{l+1}\leftarrow k_{l}↑$.  **end for** 2. Image restoration using MR-based Wiener Filter.  -Recover *I* using *k* from *B* in full-scale resolution(Equation (15))  **Output:** Blur kernel *k* and latent sharp image *I*.

## 4. Experiments

In order to prove the effectiveness of our algorithm, we compare it with several state-of-art approaches on synthetic and real blurred images. Here, we give some implementation details. Before kernel estimation, we convert all color images to grayscale ones and experimentally set the initial value of θ to 1 based on numerous experiments. The initial value of $\alpha_{s}$ is set to 0.8 times the average gradient magnitude of the coarsest version of the blurred image. The value of θ and $\alpha_{s}$ in higher levels are obtained by dividing the value of them in the previous level by 1.1. During kernel estimation, before up-sampling the blur kernel estimated from the previous level, the negative elements of the blur kernel will be set to 0 and renormalized. In Algorithm 2, the iteration time m is set to 5, empirically. We deblur each color channel respectively in the final non-blind restoration. All experiments test on a PC running MS Windows 7 64 bit version with Intel Core i5 560 M CPU, 8 GB RAM and implementation platform is MATLAB 2014a.

### 4.1. Experimental Results and Evaluation

**Figure 9:**
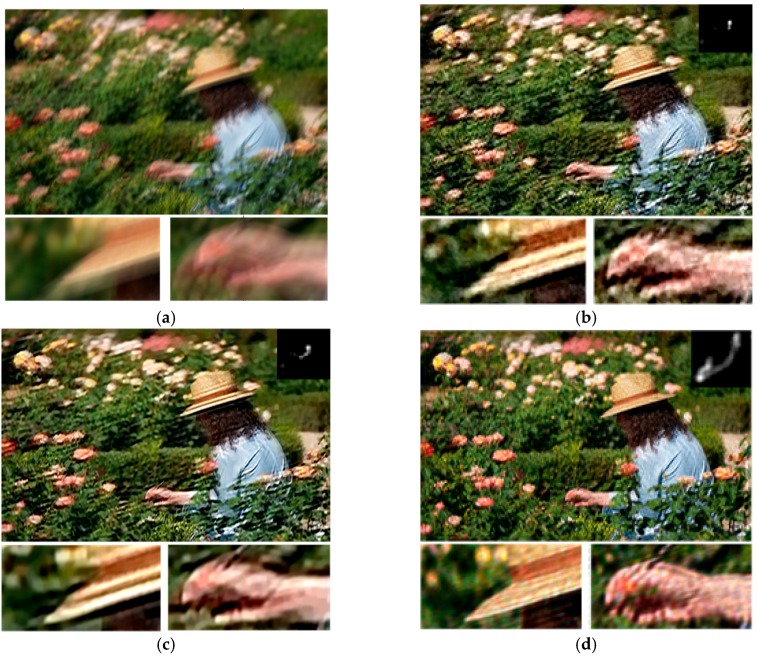

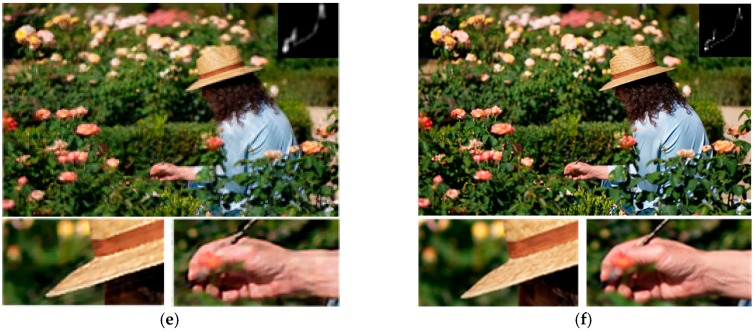
Comparison of different restoration algorithms on blur image with rich textures and small details. (**a**) Blurred image; (**b**) result of Fergus *et al.* [3]; (**c**) result of Shan *et al.* [4]; (**d**) result of Xu and Jia [7]; (**e**) our result; and (**f**) the ground truth image and blur kernel. The size of blur kernel is 27 × 27.

It is known that the more textures and small details in the blurred image, the harder the restoration. We, firstly, give a synthetic example shown in Figure 9 which contains rich textures and small details, such as flowers and leaves, to prove the robustness and effectiveness of our algorithm. The deblurred result of Fergus *et al.* [3] and Shan *et al.* [4] still contain some blur and ringing artifacts due to the inaccurate kernel estimation. The approach of Jia *et al.* [7] performs better, but the imperfect estimated kernel also leads to some artifacts in the restored result. In contrast, our algorithm gives the best performance on both the estimated blur kernel and final restored image shown in Figure 9e.

In Table 1, we adopt PSNR (Peak Signal to Noise Ratio, which is defined as Equation (17)) and SSDE to give a quantification comparison of the estimated blur kernels and deblurred images in Figure 8, respectively. As shown in Table 1, our algorithm gives a higher PSNR value for the deblurred image and lower SSDE value for the estimated blur kernel.

(17) $$PSNR\;=\;10\;\log_{10}\left\{\frac{R^{2}\;\times \;MN}{\sum_{\left(x,y\right)}{\left[I_{GT}(x,\;y)\;-\;I(x,\;y)\right]}^{2}}\right\}$$

where I and $I_{GT}$ are the restored latent sharp image and ground truth image, respectively, whose width is *M* and height is *N*. The symbol *R* denotes the maximum value of the input image data type, e.g., *R* = 255 when the images have 8-bit unsigned integer data type.

**Figure 10:**
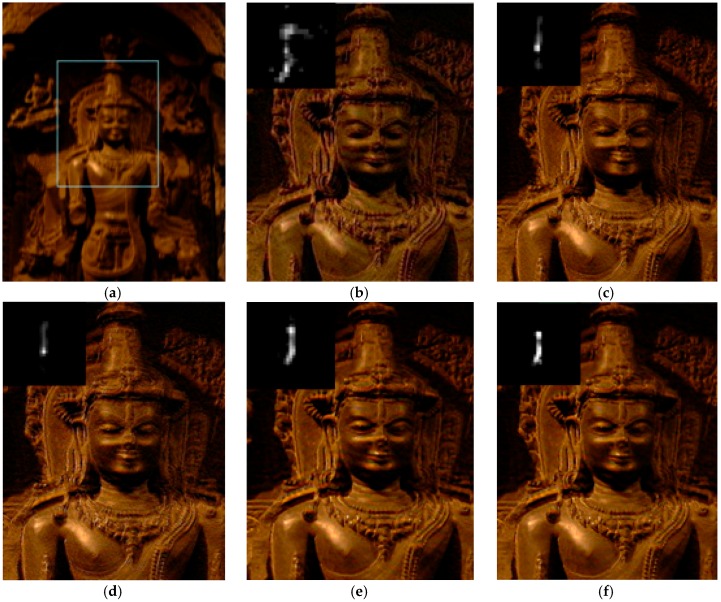
Comparison of different restoration algorithms on the Buddha image. (**a**) Real blurred image, (**b**-**f**) are the restored results of the region in the box in (**a**); (**b**) result of Fergus *et al.* [3]; (**c**) result of Shan *et al.* [4]; (**d**) result of Cho and Lee [6]; (**e**) result of Xu and Jia [7]; and (**f**) result of our algorithm.

**Figure 11:**
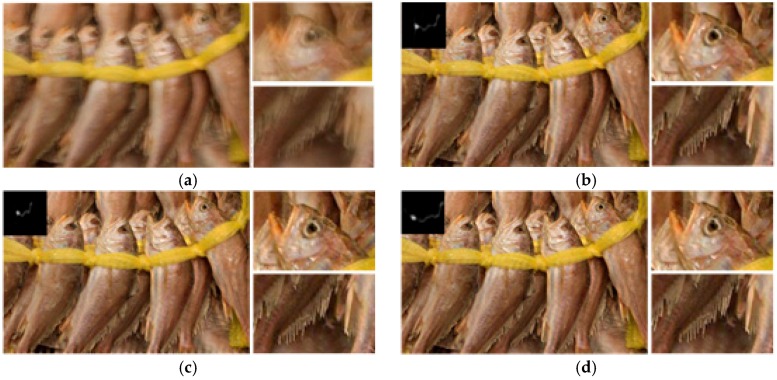
Comparison of different restoration algorithms on the fish image. (**a**) real burred image; (**b**) result of Krishnan *et al.* [13]; (**c**) result of Shan *et al.* [4]; and (**d**) our result.

**Figure 12:**
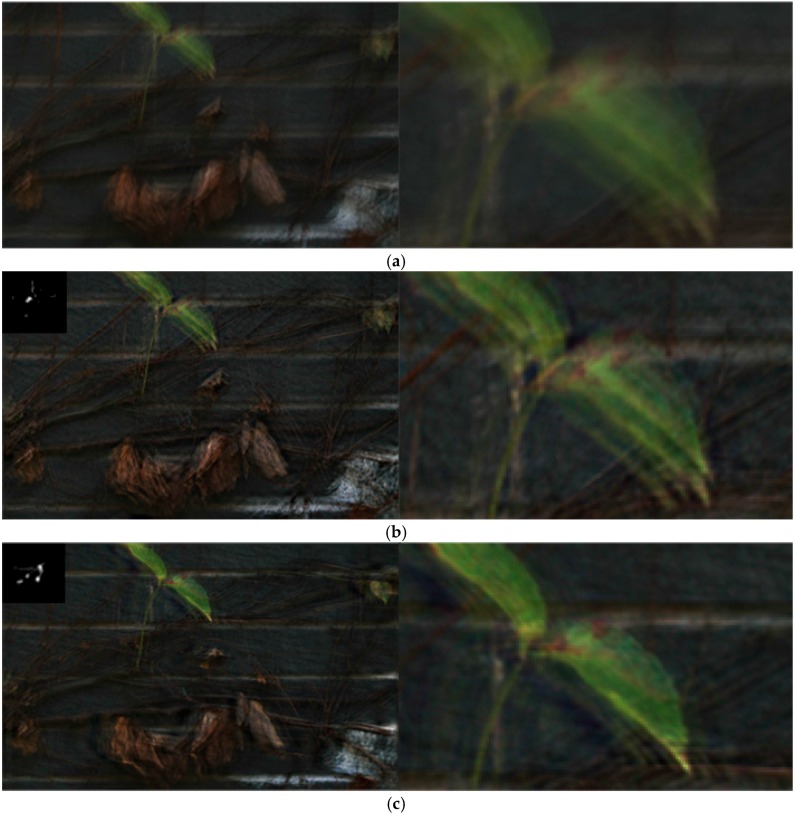

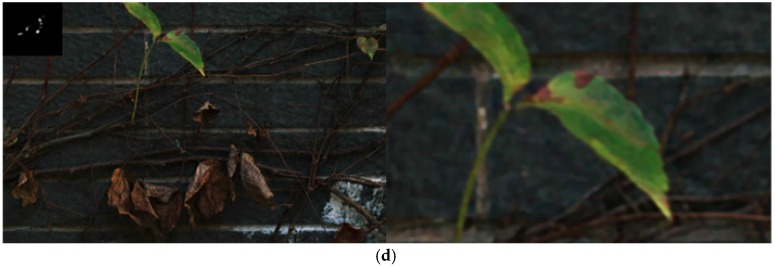
Comparison of different restoration algorithms on the wall image with large blur. (**a**) Real burred image; (**b**) result of Fergus *et al.* [3]; (**c**) result of Shan *et al.* [4]; and (**d**) our result. The size of our estimated blur kernel is 95 × 95.

We next test our algorithm on real blurred images, as shown in Figure 10, Figure 11 and Figure 12, which are presented in some previous works for comparison. Figure 10a is the real blurred image used in [3]. Due to the inaccurate blur kernel estimation, the results of [3,4,6] contain some noise. The method of [7] shows a better result, but there is still some noise in the estimated blur kernel. Our result shown in Figure 10f performs best in both kernel estimation and latent image restoration. Figure 11a is the blurred fish image used in [13]. The result of [13] contains same noise both in the estimated blur kernel and restored image. The result of [4] performs better in kernel estimation, but the restored image still contains some ringing artifacts. By contrast, our result shown in Figure 11d gives the best performance.

In addition to the capability in dealing with the blurred image containing rich textures and small details, our algorithm also can deal with large blur kernels, as shown in Figure 12. Figure 12a is the blurred wall image which contains large motion blur and small details. Due to the large blur, the method of [3] cannot estimate the blur kernel accurately and the restored latent image still contains some blur. The restored result of [4] still contains some noise and ringing artifacts. The results of our algorithm give a clearer image with finer details.

### 4.2. Operation Speed

During the restoration process, we estimated the blur kernel and latent sharp image alternately, which involved a few matrix-vector or convolution operations. For the operation speed, the MATLAB implementation of the proposed algorithm spends about 90 s to estimate a 27 × 27 blur kernel from a 255 × 255 blurred image with an Intel i5 560 M CPU@2.67 GHz and 8 GB RAM. In comparison, methods [3,13,16] need about 6 min, 3 min, and 4 min, respectively, which are computed by using the author’s MATLAB source code. The algorithm [4] implemented in C++ spends about 50 s. As the proposed algorithm involves non-convex models in kernel estimation, it needs more computational time than [6,7]. As the proposed algorithm uses a CG method and FFTs for optimization, we believe that it is feasible to accelerate by using a GPU with the strategy in [6].

## 5. Conclusions

In this paper, we propose a robust image restoration algorithm for motion blur of image sensors. Our algorithm is composed of three steps: reliable structures extraction, blur kernel estimation, and non-blind deconvolution. We implement it in a multi-scale coarse-to-fine manner. Benefiting from the self-adaptive reliable structures extraction method, the structures which have adverse effect on kernel estimation will be removed. Then we use the reliable structures of the blur image and priors of the blur kernel, such as sparsity and continuity, to estimate and refine the blur kernel. After obtaining the blur kernel, we use the fast MR-based Wiener filter to restore the final latent image. Our algorithm can deal with large blur kernels even when the blurred images contain abundant textures and small details.

However, as saturated regions in blur images destroy the linearity of the blur model, our algorithm cannot estimate the blur kernel accurately and fails to restore the blur images which contain saturated regions. Furthermore, our algorithm also cannot deal with the spatially-varying blur. Our future work is to resolve these limitation.

## Figures and Tables

**Table 1:** Comparison of the results in Figure 8.

Algorithm	[3]	[4]	[7]	Ours
PSNR of images	7.6775	9.8314	12.2182	18.7644
SSDE of kernels	0.2859	0.0434	0.0286	0.0025
